# Outcome after stroke attributable to baseline factors—The PROSpective Cohort with Incident Stroke (PROSCIS)

**DOI:** 10.1371/journal.pone.0204285

**Published:** 2018-09-26

**Authors:** Carolin Malsch, Thomas Liman, Silke Wiedmann, Bob Siegerink, Marios K. Georgakis, Steffen Tiedt, Matthias Endres, Peter U. Heuschmann

**Affiliations:** 1 Institute of Clinical Epidemiology and Biometry, University Würzburg, Würzburg, Germany; 2 Comprehensive Heart Failure Center, University of Würzburg, Würzburg, Germany; 3 Klinik und Hochschulambulanz für Neurologie, Charité - Universitätsmedizin Berlin, Berlin, Germany; 4 Center for Stroke Research Berlin, Charité - Universitätsmedizin Berlin, Berlin, Germany; 5 Institute for Stroke and Dementia Research, University Hospital of Ludwig-Maximilians-University, Munich, Germany; 6 German Center for Neurodegenerative Diseases Partner Site Berlin, Berlin, Germany; 7 German Center for Cardiovascular Research Partner Site Berlin, Berlin, Germany; 8 Berlin Institute of Health, Berlin, Germany; 9 Clinical Trial Centre Würzburg, University Hospital Würzburg, Würzburg, Germany; Medizinische Universitat Innsbruck, AUSTRIA

## Abstract

**Background:**

The impact of risk factors on poor outcome after ischemic stroke is well known, but estimating the amount of poor outcome attributable to single factors is challenging in presence of multimorbidity. We aim to compare population attributable risk estimates obtained from different statistical approaches regarding their consistency. We use a real-life data set from the PROSCIS study to identify predictors for mortality and functional impairment one year after first-ever ischemic stroke and quantify their contribution to poor outcome using population attributable risks.

**Methods:**

The PROSpective Cohort with Incident Stroke (PROSCIS) is a prospective observational hospital-based cohort study of patients after first-ever stroke conducted independently in Berlin (PROSCIS-B) and Munich (PROSCIS-M). The association of baseline factors with poor outcome one year after stroke in PROSCIS-B was analysed using multiple logistic regression analysis and population attributable risks were calculated, which were estimated using sequential population attributable risk based on a multiple generalized additive regression model, doubly robust estimation, as well as using average sequential population attributable risk. Findings were reproduced in an independent validation sample from PROSCIS-M.

**Results:**

Out of 507 patients with available outcome information after 12 months in PROSCIS-B, 20.5% suffered from poor outcome. Factors associated with poor outcome were age, pre-stroke physical disability, stroke severity (NIHSS), education, and diabetes mellitus. The order of risk factors ranked by magnitudes of population attributable risk was almost similar for all methods, but population attributable risk estimates varied markedly between the methods. In PROSCIS-M, incidence of poor outcome and distribution of baseline parameters were comparable. The multiple logistic regression model could be reproduced for all predictors, except pre-stroke physical disability. Similar to PROSCIS-B, the order of risk factors ranked by magnitudes of population attributable risk was almost similar for all methods, but magnitudes of population attributable risk differed markedly between the methods.

**Conclusions:**

Ranking of risk factors by population impact is not affected by the different statistical approaches. Thus, for a rational decision on which risk factor to target in disease interventions, population attributable risk is a supportive tool. However, population attributable risk estimates are difficult to interpret and are not comparable when they origin from studies applying different methodology. The predictors for poor outcome identified in PROSCIS-B have a relevant impact on mortality and functional impairment one year after first-ever ischemic stroke.

## Introduction

A number of studies identify prognostic factors for death or poor functional outcome after stroke [1–5]. Most studies report these associations using rate ratios, odds ratios or hazard ratios, hereby estimating risk increase for exposed compared to non-exposed patients. Only few studies take into account the prevalence of these prognostic factors in the stroke population [3–7]. Development of prevention programs, however, requires considering the prevalence of risk factors and their impact on outcome to prioritize the best targets to reduce mortality and morbidity.

Population attributable risk (PAR), as a function of risk factor prevalence and relative risk, is used to calculate the amount of poor outcome that is attributable to a prognostic factor [8, 9]. The few recent studies on mortality and functional outcome after ischemic stroke providing PAR estimates have used diverse estimation approaches [3, 5–7]. It is known that the use of diverse methodology affects the magnitudes of the resulting estimates [10], which restricts the comparability of PAR estimates from different studies applying different methodologies.

In this study, we aim to compare PAR estimates obtained from different statistical approaches regarding their consistency to evaluate the impact of the underlying statistical model on the PAR estimates. This is the first study that simultaneously assesses and directly compares PAR values obtained by different methodologies. We use a real-life data set from the PROSCIS study to identify baseline prognostic factors for death or functional impairment one year after first-ever ischemic stroke and assess the extent to which each prognostic factor contributes to poor outcome. We validate the findings within an independent data set.

## Materials and methods

The data that support the findings of this study are available to all interested researchers at Harvard Dataverse (https://doi.org/10.7910/DVN/REBNRX).

### The PROSCIS study

The Prospective Cohort with Incident Stroke (PROSCIS) is a prospective, observational, hospital-based cohort study of patients with first-ever stroke conducted independently at two centers in Germany (PROSCIS-B: Center for Stroke Research Berlin, Charité University Hospital, ClinicalTrials.gov identifier: NCT01363856; PROSCIS-M: Institute for Stroke and Dementia Research, Klinikum der Universität München, Ludwig-Maximilians-University, ClinicalTrials.gov identifier: NCT01364168). Details have been published previously [11]. Briefly, patients with ischemic stroke, primary hemorrhage or sinus venous thrombosis were recruited for PROSCIS-B at stroke units of three tertiary care university hospitals at the Charité - Universitätsmedizin Berlin since March 2010. For PROSCIS-M, patients with ischemic stroke and documented duration of disturbance of cerebral function were recruited since March 2011. Identical core protocols and data collection methods were applied by both centers, allowing studies to serve as a validation sample for each other. The main objective of PROSCIS is to determine prediction models of different complexity for the combined vascular end-point of stroke, myocardial infarction, and vascular death at three-years after first-ever stroke [11]. Patients were interviewed within the first seven days after symptom onset. An extensive clinical examination was conducted, and functional outcome was documented. Stroke survivors were followed-up annually for three years after recruitment by telephone interviews assessing i.a. patient's vital status, mood and cognitive function. Information on vital status was complemented by contacting the registry office, if patients were lost to follow-up.

### Study population

Patients 18 years or older who suffered from stroke according to the WHO criteria were included into the PROSCIS-B cohort. Exclusion criteria were prior stroke and participation in an intervention study. We restrict our analyses to patients with ischemic stroke and known survival status and functional outcome one year after stroke. A subsample of 200 patients randomly selected from the PROSCIS-M cohort serves as an external validation data set.

### Patient characteristics

Factors that are already known to have an impact on poor outcome after stroke and that were collected at baseline in the PROSCIS-B study were analyzed: sociodemographic parameters [age, sex, graduation (no graduation/ ≤10 years/ >10 years school attendance), education (total number of years spent in general, professional or university training), migration background (both parents or at least one parent and the patient are foreign-born), institutionalization pre-stroke (living in care home, retirement home or assisted living)], stroke related risk factors [body mass index (BMI), active smoking (current smoking vs. never or former smoking), regular alcohol consumption (≥20cl of wine/champagne or 50cl of beer or 2cl of hard liquor at least once per week), degree of physical activity pre-stroke (no / sparse / 1-2x20 minutes strong / ≥3x20 minutes strong physical activity), physical disability, hypertension, dyslipidemia, diabetes mellitus type I or II (DM), atrial fibrillation (AF), myocardial infarction (MI) or angina pectoris (AP), transient ischemic attack (TIA), peripheral arterial disease (PAD)], etiologic subtype of ischemic stroke according to the Trial of ORG 10172 in Acute Stroke Treatment (TOAST) classification [12] and stroke severity according to National Institute of Health Stroke Scale (NIHSS) [13, 14].

### Poor outcome one year after stroke

Poor outcome was defined as death or functional impairment (modified Rankin Scale (mRS)>2 or Barthel Index (BI)<60) one year after ischemic stroke [15–17]. Barthel Index and modified Rankin Scale were validated for German language [14, 18] and for the use in telephone interviews [18, 19].

### Statistical analysis

Univariable binary logistic regression analysis was conducted to model the chance of poor outcome comparing exposed to non-exposed individuals. Parameters with univariable p-values <0.1 were transferred to multiple binary logistic regression analysis with Firth’s maximum likelihood penalization method and backward selection (α_stay_ = 0.05) to select predictors for further analysis in order to identify the strongest and mutually independent risk factors while keeping the number of covariables to the minimum. Firth’s likelihood penalization method [20] was used to reduce small sample bias by applying the R package *logistf* [21]. Multicollinearity was examined using variance inflation factor statistics.

PAR were estimated using Coughlin et al.’s algorithm [22], doubly robust estimation [23] and average PAR [8, 24]. To apply PAR software, non-dichotomous independent variables needed dichotomization before estimation, which was conducted as follows: age <75 vs. ≥75 (based on [3, 4]), education (total number of years of education ≤10 vs. > 10 years, based on [25]) and NIHSS ≤4 vs. >4 (minor vs. moderate or major stroke, based on [3]).

Analyses were carried out in R software environment for statistical computing and graphics, Version 3.5.1.

### Ethics approval

The study was approved by the ethics committee of the Charité - Universitätsmedizin Berlin (EA1/218/09) and the ethics committee of the Ludwig-Maximilians-University Munich (project 366–10, 20.12.2010). Trained physicians of the trial teams carried out recruitment of patients. Patients or their legal representative gave written informed consent for study participation. Patients had to be awake and responsive to be enrolled into the trial.

Consent for publication of raw data was not obtained from the participants. We have followed guidelines on preparing clinical data for publication [26]. Resultantly, we have blocked the indirect identifiers sex and BMI, and dichotomized the further indirect identifiers age and education, in order to preserve the privacy of the participants. The dataset is fully anonymous. Publication of the dataset clearly and obviously presents minimal risk to confidentiality of study participants.

## Results

### Study population

**Fig 1:**
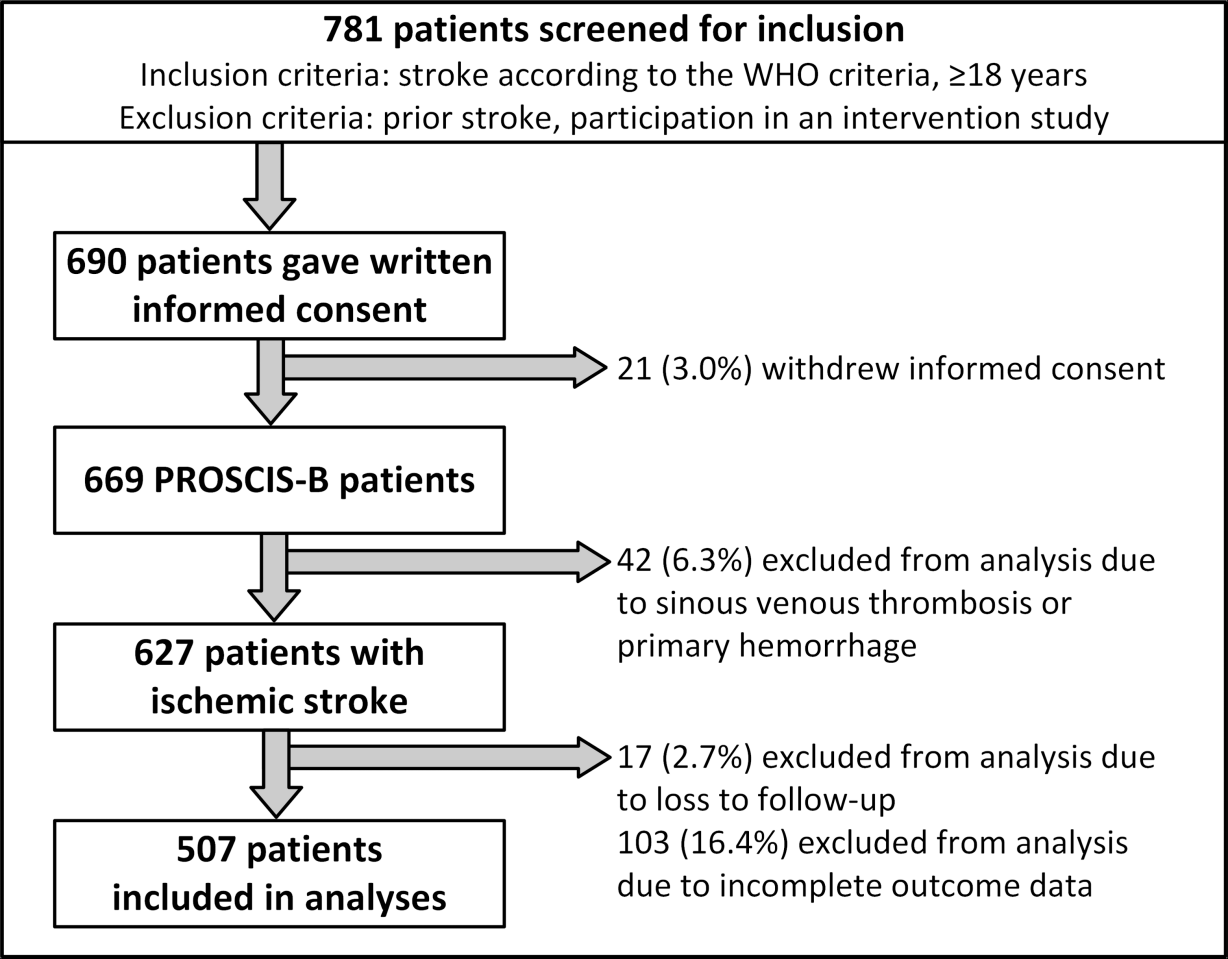
Flow chart of the study population of the PROSCIS-B cohort.

Between March 2010 and May 2013, 690 patients were recruited for PROSCIS-B. Of these, 627 patients were included in the study due to ischemic stroke. Thereof, 17 (2.7%) patients were lost to follow-up and 103 (16.4%) were alive, but had incomplete data on mRS or BI at one year. Overall, 507 (80.9%) patients were available for our analyses. Complete patient flow is shown in Fig 1.

### Patient characteristics

Patient characteristics at baseline are shown in Table 1. Within one year, 24 (4.7%) patients died and 80 (15.8%) reported relevant functional impairment. Overall, 104 (20.5%) patients had poor outcome one year after stroke and 403 (79.5%) patients were alive and did not have severe functional impairment (S1 Table). Categorization of age and NIHSS was oriented towards [3, 4, 25], and categorization of BMI towards [27]. Patients included in our analyses had a lower degree of pre-stroke physical activity, a lower rate of hypertension, higher smoking rates and lower NIHSS on admission, compared to patients excluded from analyses due to loss to follow-up or missing data on functional impairment after 12 months (S2 Table).

**Table 1:** PROSCIS-B: Patient characteristics at baseline and unadjusted associations with outcome one year after stroke assessed by univariable logistic regression analysis.

	Poor outcome at one year		Univariable analysis	
	Yes 104 (20.5%)	No 403 (79.5%)	OR (95%-CI)	p-value
**Sociodemographic parameters**				
Age, yr. mean±sd	73.6±10.6	64.9±13.2	1.06 (1.04–1.09)	<0.001
Age groups				
<65	22 (21.2%)	180 (44.7%)	1	
65–74	31 (29.8%)	130 (32.3%)	1.94 (1.08–3.51)	
75–84	34 (32.7%)	74 (18.4%)	3.72 (2.06–6.82)	
≥85	17 (16.3%)	19 (4.7%)	7.20 (3.30–15.85)	
Female sex	52 (50.0%)	145 (36.0%)	1.77 (1.15–2.74)	0.010
Graduation				<0.001
no graduation	8 (8.2%)	16 (4.1%)	5.25 (1.89–14.21)	
≤10 years of attendance	77 (78.6%)	238 (60.9%)	3.31 (1.84–6.37)	
>10 years of attendance	13 (13.3%)	137 (35.0%)	1	
Years of education, median (IQR)	12 (10–15)	13 (12–17)	0.86 (0.81–0.92)	<0.001
Migration background	11 (12.9%)	30 (8.3%)	1.67 (0.78–3.36)	0.18
Institutionalization pre-stroke	2 (1.9%)	7 (1.7%)	1.29 (0.24–4.92)	0.74
**Stroke risk factors pre-stroke**				
BMI in kg/m^2^, mean±sd	28.3±6.2	27.4±4.8	1.03 (0.99–1.08)	0.11
BMI groups in kg/m^2^				0.14
<25	34 (34.3%)	143 (35.6%)	1	
25–29.9	34 (34.3%)	170 (42.3%)	0.84 (0.50–1.42)	
≥30	31 (31.3%)	89 (22.1%)	1.46 (0.84–2.54)	
Active smoking	21 (20.4%)	110 (27.6%)	0.68 (0.40–1.13)	0.14
Regular alcohol consumption	26 (25.5%)	152 (39.0%)	0.54 (0.33–0.87)	0.011
Degree of physical activity				0.002
no physical activity	31 (30.1%)	78 (19.5%)	1	
sparse physical activity	53 (51.5%)	170 (42.6%)	0.78 (0.47–1.32)	
1-2x20 minutes strong physical activity	11 (10.7%)	84 (21.1%)	0.40 (0.16–0.70)	
≥3x20 minutes strong physical activity	8 (7.8%)	67 (16.8%)	0.31 (0.13–0.69)	
Physical disability	40 (38.5%)	53 (13.5%)	4.01 (2.46–6.53)	<0.001
Hypertension	74 (71.2%)	245 (60.8%)	1.58 (1.00–2.54)	0.051
Dyslipidaemia	21 (25.9%)	85 (25.6%)	1.03 (0.58–1.76)	0.92
Diabetes mellitus type I or II	35 (33.7%)	74 (18.4%)	2.26 (1.40–3.62)	0.001
Atrial fibrillation	40 (38.5%)	72 (17.9%)	2.87 (1.79–4.58)	<0.001
Myocardial infarction or angina pectoris	27 (26%)	54 (13.4%)	2.28 (1.34–3.81)	0.003
Transient ischemic attack	6 (6.1%)	8 (2.1%)	3.07 (1.03–8.73)	0.044
Peripheral arterial disease	14 (13.5%)	17 (4.2%)	3.54 (1.68–7.36)	0.001
**Clinical characteristics**				
Etiologic subtype of ischemic stroke				0.026
Large artery atherosclerosis	26 (25.0%)	113 (28.0%)	1	
Cardiac embolism	38 (36.5%)	84 (20.8%)	1.95 (1.11–3.48)	
Small artery occlusion	14 (13.5%)	61 (15.1%)	1.01 (0.49–2.03)	
Stroke of another determined cause	3 (2.9%)	15 (3.7%)	0.97 (0.24–3.03)	
Stroke of undetermined cause	23 (22.1%)	130 (32.3%)	0.77 (0.42–1.42)	
NIHSS, median (IQR)	3 (2–7)	2 (1–4)	1.15 (1.09–1.22)	<0.001
NIHSS groups				<0.001
0–4	65 (62.5%)	322 (79.9%)	1	
5–15	35 (33.7%)	79 (19.6%)	2.20 (1.36–3.53)	
≥16	4 (3.8%)	2 (0.5%)	8.86 (1.92–51.71)	

NIHSS, National Institute of Health Stroke Scale; BMI, Body Mass Index; IQR, inter quartile range. Analyses were restricted to patients without missing values in the respective variable.

### Logistic regression analysis

**Fig 2:**
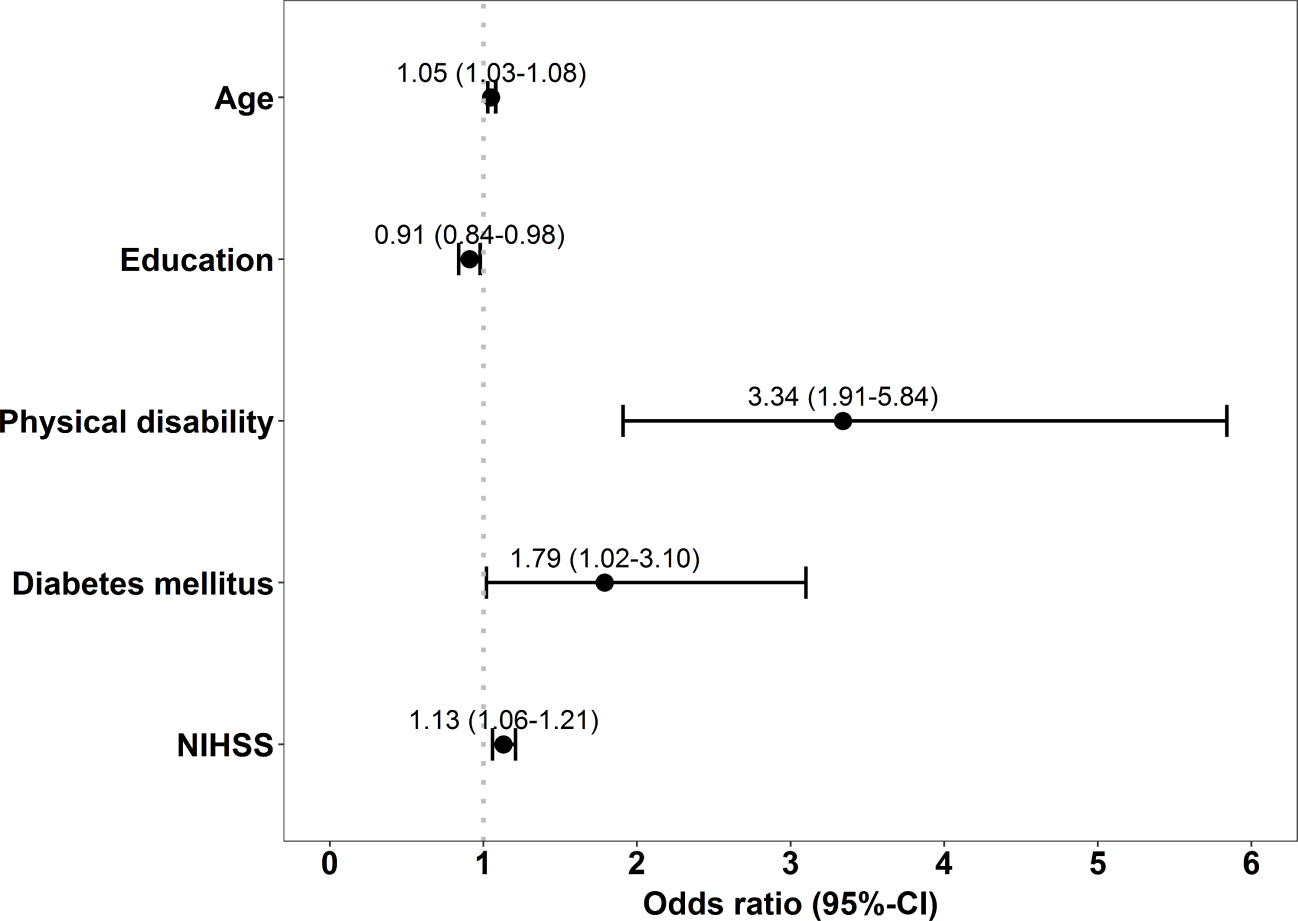
Odds ratios and respective confidence intervals gained by multiple binary logistic regression analysis after backward selection.

**Fig 3:**
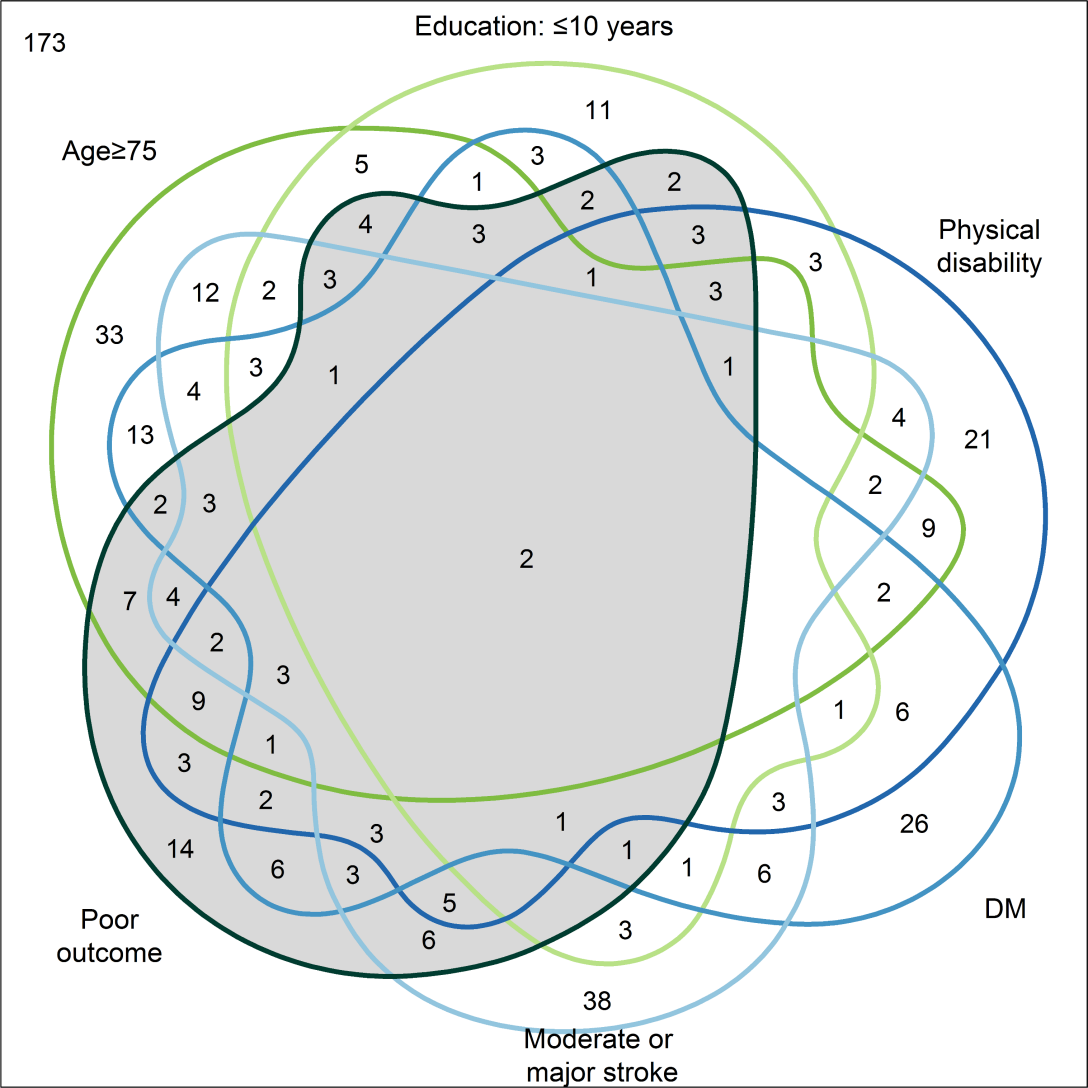
Venn diagram illustrating the intersections of the independent predictors and poor outcome 12 months after stroke.

In unadjusted logistic regression, age, sex, graduation, education, etiologic subtype of stroke, NIHSS, and pre-stroke regular alcohol consumption, degree of physical activity, physical disability, hypertension, DM, AF, MI/AP, TIA and PAD were associated with poor outcome (p<0.1) (Table 1). Independent predictors for poor outcome one year after first-ever ischemic stroke were age, education, NIHSS, pre-stroke physical disability and DM (Fig 2). Fig 3 shows the overlap of patients with poor outcome and the exposition to each of the risk factors in a Venn diagram. The grey-shaded area represents all patients with poor outcome. Of patients with poor outcome, 14 (13.5%) were not exposed to any of the predictors, 24 (23.1%) were exposed to exactly one risk factor, whereas 62 (59.6%) patients had multiple exposures and 4 (3.8%) patients had missing values in one of the predictors.

### Population attributable risk

Results from PAR estimation for PROSCIS-B are presented in Table 2. PAR calculated using Coughlin’s method (estimation requires regression parameters from the generalized additive regression model, which are presented in S3 Table) result in estimates almost twice the size of those gained from average PAR estimation, PAR estimates from doubly robust estimation are situated in between. Despite these differences, the ranking by magnitudes of PAR is very similar in all methods.

**Table 2:** PROSCIS-B: Population attributable risks of prognostic factors for poor outcome one year after stroke assessed by three different approaches.

	Average PAR		Doubly robust estimation		Coughlin’s PAR	
	PAR	rank	PAR	rank	PAR	Rank
**PROSCIS-B**						
Physical disability	18.48%	**(1)**	21.90%	**(1)**	28.85%	**(2)**
Age ≥75 years	17.29%	**(2)**	21.27%	**(2)**	29.63%	**(1)**
NIHSS >4 points	10.90%	**(3)**	13.44%	**(3)**	19.64%	**(3)**
Education ≤ 10 years	10.39%	**(4)**	12.67%	**(4)**	18.33%	**(4)**
Diabetes mellitus	7.61%	**(5)**	9.64%	**(5)**	14.83%	**(5)**

NIHSS, National Institute of Health Stroke Scale; PAR, population attributable risk.

### Validation

Validation of the analysis was conducted in a randomly selected subsample of 200 patients from the PROSCIS-M cohort. Thirty-nine (19.5%) patients had poor outcome one year after first-ever ischemic stroke. PROSCIS-M patients were older (69.9±13.4 vs. 66.7±13.2 years), more often female (43.0% vs. 38.9%), had more severe strokes (median NIHSS (IQR): 3 (1–5) vs. 2 (1–4)), had more often dyslipidaemia (36.0% vs. 25.7%) and TIA (6.5% vs. 2.9%), and less often DM (13.0% vs. 21.5%), AF (15.5% vs. 22.1%) and MI/AP (11.0% vs 16.0%) compared to patients from PROSCIS-B (S4 Table). Parameters associated with poor outcome in univariable analysis (p<0.1) were age, sex, graduation, education, NIHSS and pre-stroke active smoking, regular alcohol consumption, degree of physical activity, hypertension, dyslipidaemia, DM, MI/AP and PAD (S4 Table). The findings from the multiple binary logistic regression model derived in PROSCIS-B could be reproduced in PROSCIS-M, except pre-stroke physical disability. Associations with poor outcome were as follows (OR (CI), p-value): age 1.06 (1.02–1.10), p = 0.004; education 0.78 (0.65–0.91), p = 0.001; physical disability 1.36 (0.57–3.17), p = 0.48; DM 3.83 (1.38–10.79), p = 0.011 and NIHSS 1.16 (1.06–1.27), p<0.001.

Results from PAR estimation are shown in Table 3. As observed before in PROSCIS-B, the magnitudes of PAR estimates differ noticeably between the methods. Sequential PAR estimates from Coughlin’s approach are considerably higher compared with estimates gained from average PAR estimation and PAR estimates from doubly robust estimation are situated in between. Despite these differences, the ranking by magnitudes of PAR is similar in all methods.

**Table 3:** PROSCIS-M: Population attributable risks of prognostic factors for poor outcome one year after stroke assessed by three different approaches.

	Average PAR		Doubly robust estimation		Coughlin’s PAR	
	PAR	rank	PAR	rank	PAR	Rank
Physical disability	2.89%	**(5)**	4.28%	**(5)**	6.36%	**(5)**
Age ≥75 years	20.89%	**(2)**	28.33%	**(2)**	38.91%	**(2)**
NIHSS >4 points	26.13%	**(1)**	32.64%	**(1)**	41.45%	**(1)**
Education ≤ 10 years	18.86%	**(3)**	24.78%	**(3)**	33.38%	**(3)**
Diabetes mellitus	10.03%	**(4)**	13.42%	**(4)**	19.87%	**(4)**

NIHSS, National Institute of Health Stroke Scale; PAR, population attributable risk

However, the impact of risk factors on poor outcome differs between PROSCIS-B and PROSCIS-M, attributing the highest amount of poor outcome to physical disability and age≥75 years in PROSCIS-B and to NIHSS>4 and age≥75 years in PROSCIS-M.

## Discussion

The planning of an intervention study requires knowledge of the approximate amount of poor outcome attributable to the risk factor at target. PAR is a methodological tool to prioritize targets for modification according to their assumed contribution to reduce the outcome of interest, in our case patient’s mortality and morbidity. While different estimation methods were used in previous studies [3, 5–7], to the best of our knowledge, this is the first publication that simultaneously assesses and directly compares PAR values obtained by different statistical approaches in a real-life data set. In our analysis, the ranking by magnitudes of PAR was almost similar for all methods. However, we observed a relevant variation of PAR values gained by different estimation methods. Average PAR estimation yielded the smallest PAR values and Coughlin et al.’s approach yielded the highest estimates of PAR, being the latter roughly twice the size of the average PAR. The reason for this variation is probably the stepwise elimination approach of the sequential procedures (Coughlin et al.’s approach and doubly robust estimation), resulting in dependency of PAR estimates on the order of elimination of the risk factors from the population: based on confounder-adjusted relative risks, PAR for one risk factor is obtained by identifying the excess amount of poor outcome in exposed individuals and remove it from the population [8]. For the next risk factor, the procedure is repeated. To obtain PAR values for each risk factor from a multivariable model, the sequential procedure starts with the risk factor of interest being eliminated at first. Hence, the result is the proportion of poor outcome that can be prevented, if each risk factor is eliminated from the population at first. Especially in cases of multimorbidity, however, poor outcome can often be attributed to more than one factor and is then assumed to be eliminated multiple times. A large proportion of patients with poor outcome in PROSCIS-B (grey-shaded area in Fig 3) suffered from multiple risk factors, i.e. has multiple options to prevent poor outcome. This causes a relevant source of over-estimation of PAR when applying sequential PAR estimation methods. Coughlin’s approach seems more vulnerable to multimorbidity than average PAR, i.e. yields notablely higher estimates. By contrast, average PAR is obtained by calculating sequential PAR for every possible order of risk factor removal from the population, and subsequently averages the results for each risk factor [8]. As a result, average PAR is independent from the order of risk factor removal. Therefore, PAR values gained by different approaches are not directly comparable, in particular in scenarios where multimorbidity is present.

Furthermore, PAR values depend on the definition of exposure categories, since the application of software for PAR estimation required dichotomization of the prognostic factors. Consideration of continuous factors or factors with multiple categories was not possible. In addition, elimination of all expositions from the population is unrealistic and hence, the PAR can only be interpreted as the in theory maximum avoidable amount of poor outcome that might be preventable, if in the best scenario all expositions could be eliminated from the population.

In the present study, we estimated the population impact of known risk factors for death or functional impairment one year after ischemic stroke in a large prospective cohort (PROSCIS-B) and validated the findings in an independent cohort (PROSCIS-M). Both cohorts were designed identically, applied the same study core protocol, and the same statistical approaches were used for analysis. The associations determined from the validation cohort PROSCIS-M were similar to those from PROSCIS-B, except pre-stroke physical disability. Although we observed nominal higher probabilities in disabled compared to non-disabled patients for death (10.7% vs. 4.9%, respectively), but not for dependency (12.0% vs. 14.6%, respectively), no statistically significant associations could be observed in univariable or in multivariable analysis. This fact is probably caused by the small sample size of PROSCIS-M. The effects of age, education and NIHSS were on the same range as observed in PROSCIS-B. The effect of diabetes mellitus pointed in the same direction, but was estimated higher with less precision. The PAR magnitudes for age≥75 years (17.29% vs. 20.89%), and DM (7.61% vs. 10.03%) were comparable between both cohorts. The size of the estimated PAR values was different for physical disability pre-stroke (average PAR 18.48% vs. 2.89%), NIHSS>4 (10.90% vs. 26.13%) and education≤10 years (10.39% vs. 18.86%) for PROSCIS-B and PROSCIS-M. This might be a result of the heterogeneity of both cohorts: in PROSCIS-M, pre-stroke physical disability was less prevalent compared to PROSCIS-B and no significant association with poor outcome was found. In addition, patients in PROSCIS-B had less severe strokes and were younger, which possibly explains the notably smaller amount of poor outcome attributable to NIHSS. Moreover, patients from PROSCIS-M were better educated, which at least partly explains the higher impact of education on poor outcome. All prognostic factors for death or functional impairment after first-ever ischemic stroke in our cohort are in line with those found in previous studies [1–5, 28–32]. Furthermore, we found similar magnitudes of average PAR for age≥75 years (average PAR in PROSCIS-B: 17.29%, PROSCIS-M 20.89%), compared to previous studies applying the same PAR estimation methodology [3, 4]. Values of average PAR for DM varied between previous studies [3, 4], we found an average PAR of 7.61% in PROSCIS-B and 10.03% in PROSCIS-M. NIHSS>4 attributed 27.5% in [3], 26.13% in PROSCIS-M, but only 10.90% in PROSCIS-B. PAR of physical disability was attributable for between 9% and 17% of death and poor outcome, respectively, in [4], and for 11% and 15% in [3], which suggests that the PAR gained from PROSCIS-M might have been under-estimated. Educational level was not considered in previous average PAR estimations, but is a known risk factor for poor outcome [25]. Comparability of PAR estimates with estimates gained in previous studies, however, might still be limited, since different definitions of poor outcome were used, and the amount and types of risk factors considered varied. In [4], Barthel Index was not considered in the functional assessment, and in [3], rehospitalization due to stroke was included in the definition of poor outcome. Both studies investigated early complications in addition to sociodemographic and clinical characteristics, and results were reported stratified for mortality and poor outcome within 7 days and 3 months after stroke, respectively.

Some limitations of our study have to be considered. First, the study represents a secondary analysis of a prospective study originally designed to develop prediction models for recurrent vascular events in ischemic stroke patients. Thus, we only considered factors on hospital admission for analyses and no in-depth information on pre-stroke patient conditions, in-hospital treatment or comorbidities beyond cardiovascular diseases were available. Hence, we cannot exclude that residual confounding might be present in this study, possibly leading to an over-estimation of PAR. Nevertheless, the comprehensive and well-documented data collection process as well as the standardized conduction of all examinations helped countering information bias for the collected data. Second, the recruitment was regionally restricted to patients treated only in stroke units in Berlin and regional conditions might have had influence on the study collective, which may impede the generalizability the results. Third, we have observed a selection of study patients to less severe strokes within PROSCIS-B (median baseline NIHSS of 2 points). This might be caused by several factors: (1) recruitment took place in stroke units only, excluding more severe strokes treated at neurological intensive care units, (2) only patients able to consent or with a legal guardian could be enrolled into the study, (3) patients with available outcome after 12 months had lower values of NIHSS compared to patients with unavailable outcome information. This selection probably comes along with selective clinical characteristics such as young age, less comorbidity, and lower rates of cardio-embolic strokes as well as rather good outcomes one year after stroke and according to this, the PAR estimates from this population can only be extrapolated to similar minor stroke populations. Fourth, due to the low incidence of poor outcome of 104 out of 507 patients we used a covariable selection procedure to reduce the number of variables in the model. However, this could potentially have led to residual confounding, as we might not have enough power to detect an association of the classical risk factors like atrial fibrillation, hypertension or transient ischemic attack with poor outcome, possibly leading to an over-estimation of PAR.

To conclude, PAR is a supportive tool to make a rational decision on which risk factor to target in intervention studies. Ranking of risk factors regarding their population impact by magnitudes of PAR was independent of the estimation method. However, PAR values are difficult to interpret and are not comparable when they origin from studies applying different methodology.

## Supporting information

S1 Table Frequency of poor outcome one year after stroke. (DOCX)

S2 Table PROSCIS-B: Comparison of baseline factors between patients included into and excluded from analysis. (DOCX)

S3 Table PROSCIS-B: Association of prognostic factors with composite endpoint: Results from a multiple generalized additive regression model. (DOCX)

S4 Table PROSCIS-M: Patient characteristics at baseline and unadjusted associations with outcome one year after stroke assessed by univariable binary logistic regression analysis. (DOCX)
