# Supplementary material for: Outcome after stroke attributable to baseline factors—The PROSpective Cohort with Incident Stroke (PROSCIS)
Source: PLoS One. 2018 Sep 26;13(9):e0204285. doi: 10.1371/journal.pone.0204285 (PMC6157870; doi:10.1371/journal.pone.0204285)
Supplement: S1 Table — (DOCX) [file pone.0204285.s001.docx]

**S1 Table. Frequency of poor outcome one year after stroke.**

|  | PROSCIS-B  N=507 | PROSCIS-M  N=200 |
| --- | --- | --- |
| Death or dependency | 104 (20.5%) | 39 (19.5%) |
| Death | 24 (4.7%) | 13 (6.5%) |
| Dependency | 80 (15.8%) | 26 (13.0%) |
| Modified Rankin Scale>2 | 79 (15.6%) | 25 (12.5%) |
| Bartel Index<60 | 15 (3.0%) | 7 (3.5%) |
