# Supplementary material for: Outcome after stroke attributable to baseline factors—The PROSpective Cohort with Incident Stroke (PROSCIS)
Source: PLoS One. 2018 Sep 26;13(9):e0204285. doi: 10.1371/journal.pone.0204285 (PMC6157870; doi:10.1371/journal.pone.0204285)
Supplement: S2 Table — (DOCX) [file pone.0204285.s002.docx]

**S2 Table.** **PROSCIS-B:** **Comparison of baseline factors between patients included into and excluded from analysis.** Patients excluded from analysis are patients with ischemic stroke who participated in the PROSCIS-B Study, but were lost to follow up or had incomplete data on modified Rankin Scale or Barthel Index 12 months. NIHSS, National Institute of Health Stroke Scale; BMI, Body Mass Index; IQR, inter quartile range.

|  | **Study**  **population**  **(N=507)** | **Patients**  **Excluded**  **(N=120)** | **p-value** |
| --- | --- | --- | --- |
| **Sociodemographic parameters** |  |  |  |
| Age, yr. mean ± sd | 66.7±13.2 | 67.8±13.5 | 0.49 |
| Age groups |  |  | 0.63 |
| <65 | 202 (39.8%) | 48 (40.0%) |  |
| 65-74 | 161 (31.76%) | 32 (26.7%) |  |
| 75-84 | 108 (21.3%) | 29 (24.2%) |  |
| ≥85 | 36 (7.1%) | 11 (9.2%) |  |
| Female sex | 197 (38.9%) | 47 (39.2%) | 0.99 |
| Graduation |  |  | 0.16 |
| no graduation | 24 (4.9%) | 7 (6.3%) |  |
| ≤10 years of attendance | 315 (64.4%) | 80 (72.1%) |  |
| >10 years of attendance | 150 (30.7%) | 24 (21.6%) |  |
| Years of education, median (IQR) | 13 (12-17) | 12 (11-14) | <0.001 |
| Migration background | 41 (9.2%) | 12 (11.7%) | 0.57 |
| Institutionalization pre-stroke | 9 (1.8%) | 3 (2.5%) | 0.71 |
| **Stroke risk factors pre-stroke** |  |  |  |
| BMI in kg/m², mean ± sd | 27.53±5.1 | 27.6±4.5 | 0.64 |
| BMI groups in kg/m² |  |  | 0.85 |
| <25 | 177 (35.3%) | 38 (32.8%) |  |
| 25 -<30 | 204 (40.7%) | 48 (41.4%) |  |
| ≥30 | 120 (24.%) | 30 (25.9%) |  |
| Active smoking | 131 (26.1%) | 42 (36.5%) | 0.033 |
| Regular alcohol consumption | 178 (36.2%) | 42 (35.6%) | 0.99 |
| Degree of physical activity |  |  | 0.006 |
| no physical activity | 109 (21.7%) | 40 (33.9%) |  |
| sparse physical activity | 223 (44.4%) | 49 (41.5%) |  |
| 1-2x20 minutes strong phys. act. | 95 (18.9%) | 10 (8.5%) |  |
| ≥3x20 minutes strong phys. act. | 75 (14.9%) | 19 (16.1%) |  |
| Physical disability | 93 (18.7%) | 20 (17.0%) | 0.76 |
| Hypertension | 319 (62.9%) | 90 (75.0%) | 0.017 |
| Dyslipidaemia | 106 (25.7%) | 22 (22.9%) | 0.67 |
| Diabetes mellitus type I or II | 109 (21.5%) | 29 (24.2%) | 0.61 |
| Atrial fibrillation | 112 (22.1%) | 23 (19.2%) | 0.56 |
| Myocardial infarction/ angina pectoris | 81 (16.0%) | 19 (15.8%) | 0.99 |
| Transient ischemic attack | 14 (2.9%) | 5 (4.3%) | 0.39 |
| Peripheral arterial disease | 31 (6.1%) | 11 (9.2%) | 0.32 |
| **Clinical characteristics** |  |  |  |
| Etiologic subtype of ischemic stroke |  |  |  |
| Large artery atherosclerosis | 139 (27.4%) | 30 (25%) | 0.93 |
| Cardiac embolism | 122 (24.1%) | 27 (22.5%) |  |
| Small artery occlusion | 75 (14.8%) | 21 (17.5%) |  |
| Stroke of another determined cause | 18 (3.6%) | 4 (3.33%) |  |
| Stroke of undetermined cause | 153 (30.2%) | 38 (31.67%) |  |
| NIHSS, median (IQR) | 2 (1 – 4) | 3 (2-5) | 0.032 |
| NIHSS groups |  |  | 0.099 |
| 0 – 4 | 387 (76.3%) | 83 (69.2%) |  |
| 5 – 15 | 114 (22.5%) | 37 (30.8%) |  |
| ≥16 | 6 (1.2%) | 0 (0%) |  |
