# Supplementary material for: Outcome after stroke attributable to baseline factors—The PROSpective Cohort with Incident Stroke (PROSCIS)
Source: PLoS One. 2018 Sep 26;13(9):e0204285. doi: 10.1371/journal.pone.0204285 (PMC6157870; doi:10.1371/journal.pone.0204285)
Supplement: S3 Table — (DOCX) [file pone.0204285.s003.docx]

**S3 Table. PROSCIS-B: Association of prognostic factors with composite endpoint: results from a multiple generalized additive regression model.**

|  | OR (95%-CI) | p-value |
| --- | --- | --- |
| Age≥75 yr. | 2.53 (1.54-4.16 | <0.001 |
| Education: ≤ 10 vs. > 10 years | 3.12 (1.67-5.80) | <0.001 |
| Physical disability | 3.84 (2.26-6.54) | <0.001 |
| Diabetes mellitus | 1.77 (1.03-3.05) | 0.039 |
| NIHSS>4 | 2.07 (1.23-3.49) | 0.006 |
