# Supplementary material for: Outcome after stroke attributable to baseline factors—The PROSpective Cohort with Incident Stroke (PROSCIS)
Source: PLoS One. 2018 Sep 26;13(9):e0204285. doi: 10.1371/journal.pone.0204285 (PMC6157870; doi:10.1371/journal.pone.0204285)
Supplement: S4 Table — (DOCX) [file pone.0204285.s004.docx]

**S4 Table. PROSCIS-M:** **Patient characteristics at baseline and unadjusted associations with outcome one year after stroke assessed by univariable binary logistic regression analysis.** NIHSS, National Institute of Health Stroke Scale; BMI, Body Mass Index; IQR, inter quartile range. Analyses were restricted to patients without missing values in the respective variable.

|  | **PROSCIS-M** | **Poor outcome at one year** | | **Univariable analysis** | |
| --- | --- | --- | --- | --- | --- |
|  | N=200 | **Yes**  39 (19.5%) | **No**  161 (80.5%) | **OR (95%-CI)** | **p-value** |
| **Sociodemographic parameters** |  |  |  |  |  |
| Age, yr. mean±sd | 69.9±13.4 | 77.5±10.6 | 68.0±13.3 | 1.07 (1.03-1.11) | <0.001 |
| Age groups |  |  |  |  |  |
| <65 | 63 (31.5%) | 5 (12.8%) | 58 (36.0%) | 1 | <0.001 |
| 65-74 | 52 (26.0%) | 9 (23.1%) | 43 (26.7%) | 2.32 (0.76-7.61) |  |
| 75-84 | 66 (33.0%) | 15 (38.5%) | 51 (31.7%) | 3.20 (1.19-9.89) |  |
| ≥85 | 19 (9.5%) | 10 (25.6%) | 9 (5.6%) | 11.76 (3.55-43.59) |  |
| Female sex | 86 (43.0%) | 27 (69.2%) | 59 (36.6%) | 3.79 (1.84-8.19) | <0.001 |
| Graduation |  |  |  |  |  |
| no graduation | 0 (0%) | 0 (0%) | 0 (0%) | - |  |
| ≤10 years of attendance | 31 (15.5%) | 16 (41.0%) | 15 (9.3%) | 6.64 (2.94-15.24 | <0.001 |
| >10 years of attendance | 169 (84.5%) | 23 (59.0%) | 146 (90.7%) | 1 |  |
| Years of education, median (IQR) | 13 (11-16) | 11 (9.5-12) | 13 (12-16) | 0.71 (0.60-0.83) | <0.001 |
| Migration background | 30 (15.0%) | 5 (12.8%) | 25 (15.5%) | 0.85 (0.29-2.17) | 0.75 |
| Institutionalization pre-stroke | 4 (2.0%) | 1 (2.6%) | 3 (1.9%) | 1.76 (0.17-11.10) | 0.58 |
| **Stroke risk factors pre-stroke** |  |  |  |  |  |
| BMI in kg/m², mean ± sd | 26.0±4.2 | 25.8±4.9 | 26.0±4.1 | 0.99 (0.91-1.08) | 0.89 |
| BMI groups in kg/m² |  |  |  |  |  |
| <25 | 78 (39.0%) | 18 (46.2%) | 60 (37.3%) | 1 | 0.62 |
| 25- 29.9 | 92 (46.0%) | 16 (41.0%) | 76 (47.2%) | 0.71 (0.33-1.49) |  |
| ≥30 | 30 (15.0%) | 5 (12.8%) | 25 (15.5%) | 0.71 (0.23-1.94) |  |
| Active smoking | 41 (20.5%) | 4 (10.3%) | 37 (23.0%) | 0.42 (0.13-1.10) | 0.079 |
| Regular alcohol consumption | 150 (75.0%) | 25 (64.1%) | 125 (77.6%) | 0.51 (0.25-1.09) | 0.082 |
| Degree of physical activity |  |  |  |  | 0.008 |
| no physical activity | 53 (26.5%) | 16 (41.0%) | 37 (23.0%) | 1 |  |
| sparse physical activity | 63 (31.5%) | 16 (41.0%) | 47 (29.2%) | 0.79 (0.35-1.77) |  |
| 1-2x20 minutes strong physical activity | 37 (18.5%) | 3 (7.7%) | 34 (21.1%) | 0.23 (0.06-0.73) |  |
| ≥3x20 minutes strong physical activity | 47 (23.5%) | 4 (10.3%) | 43 (26.7%) | 0.24 (0.07-0.68) |  |
| Physical disability | 56 (28.0%) | 12 (30.8%) | 44 (27.3%) | 1.20 (0.55-2.51) | 0.64 |
| Hypertension | 117 (58.5%) | 29 (74.4%) | 88 (54.7%) | 2.33 (1.11-5.24) | 0.025 |
| Dyslipidemia | 72 (36.0%) | 20 (51.3%) | 52 (32.3%) | 2.19 (1.09-4.45) | 0.029 |
| Diabetes mellitus type I or II | 26 (13.0%) | 11 (28.2%) | 15 (9.3%) | 3.81 (1.59-9.02) | 0.003 |
| Atrial fibrillation | 31 (15.5%) | 7 (17.9%) | 24 (14.9%) | 1.30 (0.50-3.08) | 0.58 |
| Myocardial infarction or angina pectoris | 22 (11.0%) | 9 (23.1%) | 13 (8.1%) | 3.43 (1.34-8.53) | 0.011 |
| Transient ischemic attack | 13 (6.5%) | 1 (2.6%) | 12 (7.5%) | 0.47 (0.05-2.03) | 0.34 |
| Peripheral arterial disease | 13 (6.5%) | 7 (17.9%) | 6 (3.7%) | 5.52 (1.80-17.44) | 0.003 |
| **Clinical characteristics** |  |  |  |  |  |
| Etiologic subtype of ischemic stroke |  |  |  |  | 0.36 |
| Large artery atherosclerosis | 39 (19.5%) | 9 (23.1%) | 30 (18.6%) | 1 |  |
| Cardiac embolism | 61 (30.5%) | 15 (38.5%) | 46 (28.6%) | 1.07 (0.43-2.78) |  |
| Small artery occlusion | 19 (9.5%) | 2 (5.1%) | 17 (10.6%) | 0.46 (0.08-1.86) |  |
| Stroke of another determined cause | 10 (5.0%) | 0 (0%) | 10 (6.2%) | 0.15 (0.00-1.40) |  |
| Stroke of undetermined cause | 71 (35.5%) | 13 (33.3%) | 58 (36.0%) | 0.74 (0.29-1.94) |  |
| NIHSS, median (IQR) | 3 (1-5.25) | 6 (3-10.5) | 2 (1-5) | 1.16 (1.08-1.25) | <0.001 |
| NIHSS groups |  |  |  |  | <0.001 |
| 0-4 | 138 (69.0%) | 18 (46.2%) | 120 (74.5%) | 1 |  |
| 5-15 | 54 (27.0%) | 16 (41.0%) | 38 (23.6%) | 2.79 (1.30-5.97) |  |
| ≥16 | 8 (4.0%) | 5 (12.8%) | 3 (1.9%) | 10.24 (2.51-47.53) |  |
